# Chronic Inactivation of the Orbitofrontal Cortex Increases Anxiety-Like Behavior and Impulsive Aggression, but Decreases Depression-Like Behavior in Rats

**DOI:** 10.3389/fnbeh.2016.00250

**Published:** 2017-01-23

**Authors:** Hiroshi Kuniishi, Satoshi Ichisaka, Sae Matsuda, Eri Futora, Riho Harada, Yoshio Hata

**Affiliations:** ^1^Division of Integrative Bioscience, Institute of Regenerative Medicine and Biofunction, Tottori University Graduate School of Medical SciencesYonago, Japan; ^2^Division of Neurobiology, School of Life Sciences, Faculty of Medicine, Tottori UniversityYonago, Japan

**Keywords:** orbitofrontal cortex, anxiety, depression, impulsive aggression, corticosterone, chronic inactivation, muscimol

## Abstract

The orbitofrontal cortex (OFC) is involved in emotional processing, and orbitofrontal abnormalities have often been observed in various affective disorders. Thus, chronic dysfunction of the OFC may cause symptoms of affective disorders, such as anxiety, depression and impulsivity. Previous studies have investigated the effect of orbitofrontal dysfunction on anxiety-like behavior and impulsive aggression in rodents, but the results are inconsistent possibly reflecting different methods of OFC inactivation. These studies used either a lesion of the OFC, which may affect other brain regions, or a transient inactivation of the OFC, whose effect may be restored in time and not reflect effects of chronic OFC dysfunction. In addition, there has been no study on the effect of orbitofrontal inactivation on depression-like behavior in rodents. Therefore, the present study examined whether chronic inactivation of the OFC by continuous infusion of a GABA_A_ receptor agonist, muscimol, causes behavioral abnormalities in rats. Muscimol infusion inactivated the ventral and lateral part of the OFC. Following a week of OFC inactivation, the animals showed an increase in anxiety-like behavior in the open field test and light-dark test. Impulsive aggression was also augmented in the chronically OFC-inactivated animals because they showed increased frequency of fighting behavior induced by electric foot shock. On the other hand, chronic OFC inactivation reduced depression-like behavior as evaluated by the forced swim test. Additionally, it did not cause a significant change in corticosterone secretion in response to restraint stress. These data suggest that orbitofrontal neural activity is involved in the regulation of anxiety- and depression-like behaviors and impulsive aggression in rodents.

## Introduction

The prefrontal cortex (PFC) is critical for cognitive function and affective response (Frith and Dolan, 1996; Roy et al., 2012). Prefrontal abnormalities have been reported in various psychiatric disorders, such as depression, anxiety disorder and personality disorder (Soloff et al., 2003; Drevets, 2007; Milad and Rauch, 2007). The orbitofrontal cortex (OFC), a ventral subregion of the PFC (Brodmann area 10, 11, 12, 47; Szczepanski and Knight, 2014) is involved in the integration of sensory information, emotional processing, decision making and behavioral flexibility (Rolls, 2004; Rempel-Clower, 2007; Schoenbaum et al., 2010). Orbitofrontal abnormalities have been implicated in many psychiatric symptoms, such as pathological anxiety, depression and impulsive aggression, as well as in the endocrine response to stress in human imaging studies (Soloff et al., 2003; Drevets, 2007; Milad and Rauch, 2007; Dedovic et al., 2009). Furthermore, orbitofrontal lesion leads to a heightened anxiety state and aggressiveness in human case studies and primate lesion studies (Grafman et al., 1986; Berlin et al., 2004, 2005; Izquierdo et al., 2005; Hahn et al., 2011; Agustín-Pavón et al., 2012; Shiba et al., 2015). Therefore, it is plausible that those psychiatric symptoms are caused by dysfunction of the OFC.

The relationship between psychiatric disorders and prefrontal dysfunction has been investigated in rodents. Previous studies have examined the possible causal relationship between OFC function and anxiety-like behavior and aggression in rodents using electrolytic or pharmacological lesion of the OFC (Kolb, 1974; Kolb and Nonneman, 1974; de Bruin et al., 1983; Lacroix et al., 2000; Rudebeck et al., 2007; Orsini et al., 2015) or pharmacological inactivation of OFC activity (Wall et al., 2004). While an increase in aggression was observed following OFC lesion (Kolb, 1974; Kolb and Nonneman, 1974; de Bruin et al., 1983; Rudebeck et al., 2007), the lesion studies failed to find an influence on anxiety-like behavior (Lacroix et al., 2000; Rudebeck et al., 2007; Orsini et al., 2015). On the other hand, acute pharmacological inactivation of the OFC induced an augmentation of anxiety-like behavior (Wall et al., 2004). This apparent discrepancy might have arisen from the difference in experimental methods. The duration of OFC dysfunction might have affected the results. Animal behavior was examined within several minutes after starting inactivation in the acute study (Wall et al., 2004), while the lesion studies (Lacroix et al., 2000; Rudebeck et al., 2007; Orsini et al., 2015) examined behavior following a period of 1 week or more after making the OFC lesion. It is possible that the animals had recovered from the effect of OFC inactivation within the following several days, and thus, the behavioral abnormality was not found in the lesion studies. In addition, in these studies the brain region affected by lesion or inactivation might not be confined to the OFC. Electric lesion, aspiration and inactivation using lidocaine would cause unintended damage to passing fibers in the OFC, and thus, the effect of lesion and inactivation might have not been restricted to the OFC. Although excitotoxic lesion spares passing fibers, cell loss in a region elicits secondary cell loss or synaptic alterations in distant regions connected anterogradely and retrogradely (Vanburen, 1963; Poduri et al., 1995). Therefore, those previous results might contain an influence of dysfunction of unspecified brain regions other than the OFC. Because psychiatric disorders are often accompanied by chronic PFC dysfunction (Bolla et al., 2003; Drevets, 2007), it is important to examine the effect of chronic inactivation restricted to the OFC on animal behavior.

In addition to these methodological problems, no previous study has determined whether OFC inactivation affects depression-like behavior and the endocrine response to stress in rodents as far as we know. Therefore, in the present study, we continuously infused a GABA_A_ receptor agonist, muscimol, into the OFC for local and chronic inactivation of OFC neural activity (Majchrzak and Di Scala, 2000) and examined the effects on the behavioral and endocrine abnormalities such as anxiety- and depression-like behaviors, impulsive aggression and plasma corticosterone levels in rats. We found that a chronic inactivation of the ventral and lateral part of the OFC increased anxiety-like behavior and impulsive aggression. On the other hand, it reduced depression-like behavior and did not influence the hormonal response to restraint stress. These results suggest that orbitofrontal neural activity is involved in the regulation of anxiety- and depression-like behaviors and impulsive aggression in rodents.

## Materials and Methods

### Animals

Thirty-five adult male Sprague-Dawley (SD) rats (postnatal day (P) 80–83 and P184–187 at surgery, Japan SLC Inc., Hamamatsu, Japan) were used. All animals were housed in groups of three animals in plastic cages (22 (length) cm × 40 (width) cm × 18.5 (height) cm) under controlled laboratory conditions (temperature: 21–24°C) with free access to food and water under a 12 h light/dark cycle (light onset at 07:00 AM). The nesting material in each cage was replaced once a week. After surgery, all animals were housed individually in plastic cages (22 cm × 32 cm × 13.5 cm). The experimental procedures met the regulations of the animal care committee of Tottori University (approval number: 16-Y-4).

### Chronic Muscimol Infusion into the OFC

**Figure 1:**
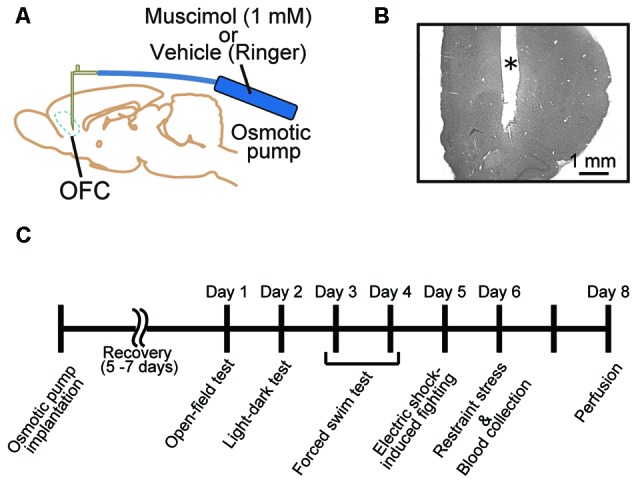
**Animal treatment and behavioral assessment. (A)** Male adult Sprague-Dawley (SD) rats were implanted with an infusion cannula and an osmotic pump for muscimol infusion to the orbitofrontal cortex (OFC) in both hemispheres. **(B)** Representative photograph of a Nissl-stained section of prefrontal cortex (PFC). *Cannula track. **(C)** Time schedule of the present experiments. A battery of behavioral tests was performed 5–7 days after the implantation. Muscimol infusion was continued until perfusion.

All surgical procedures were performed under anesthesia with 2.0%–3.0% isoflurane (Forane, Abbott, IL, USA) in O_2_. The eyes were protected against drying out with ointment (Tarivid ophthalmic ointment, Santen, Osaka, Japan). The animal was placed in a stereotaxic instrument (SR-6R-HT, Narishige, Tokyo, Japan), and the head was fixed using ear bars and a mouth bar. The scalp was cut, and lidocaine (Xylocaine Pump Spray 8%, AstraZeneca, Osaka, Japan) was applied to incisions. A small segment of skull above the OFC in both hemispheres (2.8 mm anterior to bregma and 2.0 mm lateral to midline; Paxinos and Watson, 1998) was removed. A 30 G stainless steel cannula connected to an osmotic minipump (Alzet 2002, Palo Alto, CA, USA) was implanted into the cortex in both hemispheres (4.0 mm deep from the cortical surface; Figure 1A). The exposed cortex was covered with a gelatin sponge (Spongel, Astellas Pharma Inc., Tokyo, Japan) and dental cement (ADFA, Shofu Inc., Kyoto, Japan). The scalp was sutured closed, and a local anesthetic containing antibiotics (PRONES-PASTA AROMA, Nishika, Shimonoseki, Japan) was applied to the incision. A solution containing a GABA_A_ receptor agonist, muscimol (1.0 mM in Ringer’s solution, Tocris Bioscience, Bristol, UK), was infused continuously (0.5 μl/h) to inhibit orbitofrontal cortical activity for approximately 2 weeks (*n* = 18). Vehicle (Ringer’s solution) was infused into control animals (*n* = 17). The animals were given 5–7 days for recovery prior to behavioral testing (Figure 1C).

### Behavioral Tests

Behavioral tests were conducted during the light phase of the illumination cycle. On the day of the test, rats were transported to the testing room and left in their home cages for 1 h before the test. The animals’ behavior during test trials was recorded and monitored by a PC located in the adjacent room using a universal serial bus camera and analyzed using ANY-maze^TM^ Video Tracking System (Stoelting Co., Wood Dale, IL, USA).

#### Open Field Test

##### Apparatus

The open field apparatus consisted of a square arena (70 cm × 70 cm) made of gray polyvinyl chloride plastic boards with walls of 40 cm height (Muromachi Kikai Co., Tokyo, Japan). The arena was lit by a light-emitting diode lighting placed 145 cm above the arena. The light intensity was 75 lx at the center of the arena. The test sessions were recorded by a video camera placed 145 cm above the arena.

##### Experimental procedures

To start each session, a rat was placed at a particular corner of the arena and allowed to explore for 5 min. During the test session, the total distance traveled, time in the center area (30 cm × 30 cm), and time in the thigmotaxis area (less than 10 cm away from the walls) were measured automatically using the ANY-maze^TM^ Video Tracking System. The apparatus was cleaned with 70% ethanol before the test of each animal.

#### Light-Dark Test

##### Apparatus

We performed a light-dark exploring test using a modified setup of the open field apparatus. Half of the apparatus was covered with a wooden board to divide the arena into two areas: a light side (70 cm × 35 cm, 200 lx) and a dark side (70 cm × 35 cm, 10–20 lx) with walls of 40 cm height.

##### Experimental procedures

To start each test session, a rat was placed in the light area and allowed to explore for 5 min. During the test session, the frequency of entries into the light area and the time spent in the light area were measured automatically. The apparatus was cleaned with 70% ethanol before the test of each animal. Because the light-dark test is based on the aversion of rodents to bright areas and on their spontaneous exploratory behavior in novel environments (Takao and Miyakawa, 2006), all animals moved to the dark area immediately after starting the test session, except for two animals which remained in the light area throughout the test session. These animals were excluded from the analysis to avoid the possibility that they were not normal in anxious tendency (two animals in muscimol group).

#### Forced Swim Test

##### Apparatus

The forced swim test apparatus was an acrylic cylinder (60 (height) cm × 40 (diameter) cm, Muromachi Kikai Co.) filled with water at 24 ± 1°C (depth, 40 cm). In the water, the rats could not support themselves by touching the bottom with their feet or tails. The apparatus was lit indirectly, and the light intensity was 40 lx at the surface of water.

##### Experimental procedures

The forced swim test is composed of a 15 min pretest and 5 min test performed on the next day. Following each swim session, the rats were removed from the cylinder, dried with paper towels, placed in a heated incubator for approximately 30 min, and then returned to their home cages. During the test session, immobility time was measured automatically. The duration of climbing (making vigorous upward directed movements of forelimbs) and swimming (swimming with movements of forelimbs) was counted manually. Because automatic measurement by the software judges climbing behavior as immobility, we corrected the immobility time by subtracting the duration of climbing behavior. Four animals were not included in the data analysis due to failure of video tracking (one animal in vehicle group and two animals in muscimol group).

#### Electric-Shock-Induced Fighting Test

##### Apparatus

The apparatus was constructed from an acrylic box (21 (height) cm × 20 (width) cm × 15.5 (length) cm, ENV 010-MC, Med Associates Inc., St. Albans, VT, USA). The floor of the box consisted of 0.2 cm diameter metal grids with 0.9 cm separation. Electric shocks of specified duration and intensity were delivered to the grids using equipment (SEN-7203, NIHON KOHDEN, Tokyo, Japan; ENV 414, Med Associates Inc.) located in the adjacent room.

##### Experimental procedures

The present procedure is similar to those described previously by others (Tedeschi et al., 1959; Ulrich and Azrin, 1962; Matsuoka et al., 2005). Electric-shock-induced fighting was examined by placing two rats in the box and giving them electric foot shocks (1/3 Hz, 500 ms, 1.0 mA) for 5 min. The total number and duration of fighting episodes were measured. Fighting was defined as follows according to the criteria by Ulrich and Azrin (1962): both rats face each other in an upright position, their heads thrust forward, they open their mouths, and they strike vigorously at each other.

### Restraint Stress and Blood Collection

Animals were taken from the home cage and placed into restraint tubes (KN-325-C, C-4, Natsume Seisakusho Co., Ltd., Tokyo, Japan) for 30 min. Restraint was performed between 10:00 and 16:00. Blood samples (500 μl) were obtained by making a small incision on the tail end and collected into heparinized tubes (Capiject, Terumo Medical Corp., Somerset County, NJ, USA). After the blood sampling, the animals were placed back to the home cage. Blood samples were stored at room temperature for at least 1 h and then centrifuged at room temperature for 90 s at 3500 g. Plasma was collected and stored at −80°C until the corticosterone assay.

### Corticosterone Assay

Plasma corticosterone was measured with a commercial enzyme immunoassay kit (YK240 Corticosterone EIA kit, Yanaihara Institute Inc., Fujinomiya, Japan) following the manufacturer’s protocol. The data were analyzed with Sunrise Rainbow RC analysis software (X/Fluor 4, TECAN, Männedorf, Switzerland). A standard curve was generated from the corticosterone standard of known concentration put in the same plate with the samples.

### Recording of Cortical Cell Activity

To determine the extent of muscimol infusion, cortical activity was recorded in the animals infused with muscimol or vehicle for >48 h (two vehicle-infused hemispheres and four muscimol-infused hemispheres). Both groups of animals were restrained in a stereotaxic instrument under anesthesia with 1.5%–3.0% isoflurane in O_2_ and sedation with chlorprothixene (0.5 mg/kg, i.m., Sigma-Aldrich, St. Louis, MO, USA). The body temperature was maintained at 37°C by a temperature controller (NS-TC10, NeuroScience, Tokyo, Japan). Skin was incised on the head. All incisions were infiltrated with xylocaine. A square hole (2 mm × 4 mm) was made on the skull above the OFC (stereotaxic position, anterior 2.8 mm–4.8 mm to bregma and lateral 0 mm–4.0 mm to midline). A tungsten electrode (1 MΩ, UNIQUE MEDICAL, Tokyo, Japan) was inserted at various distances and depths to the site of muscimol infusion. Neural activity was filtered at 500–5000 Hz and amplified 1000-fold by an amplifier (Model 1800, A-M systems Inc., Sequim, WA, USA). When no spontaneous activity or injury discharge was observed, we judged the site as inactivated. After recording, electrolytic lesions were made to mark the position of recording sites at two different depths by applying an electrode (−) current of 1 μA for 10 s.

### Histology

After all behavioral tests and blood collection, the animals were deeply anesthetized with isoflurane and perfused transcardially with Ringer’s solution followed by 4% paraformaldehyde in 0.1 M phosphate buffer (PB). The brains were removed and postfixed in 4% paraformaldehyde and 20% sucrose in 0.1 M PB. The brains were frozen, and coronal sections of 30 μm thickness were cut using a freezing microtome. The sections were stained with cresyl violet to determine the location of cannula tracks and electrolytic lesions (Figure 1B).

### Statistical Analysis

All statistical analyses were performed using a statistical software PASW Statistic Ver. 18 (SPSS Inc., Chicago, IL, USA). All data were analyzed using the Shapiro-Wilk test to examine the sample distribution, and statistical comparisons between two groups were carried out by unpaired *t*-test or Mann-Whitney *U*-test. Homoscedastic and heteroscedastic data were analyzed by Student’s *t*-test and Welch’s *t*-test, respectively. Statistical significance was set at *P* < 0.05.

## Results

### Electrophysiological Estimation of the Orbitofrontal Region Inactivated by Chronic Muscimol Infusion

**Figure 2:**
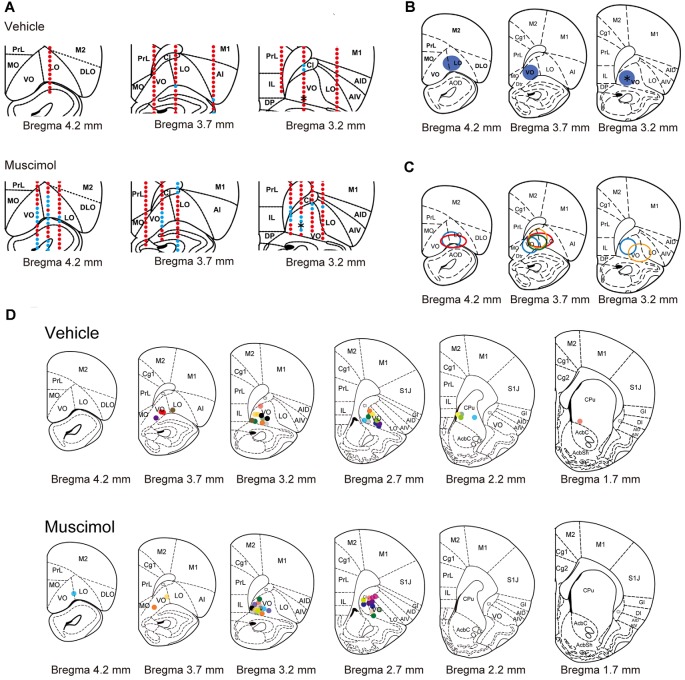
**Electrophysiological and histological estimation of the orbitofrontal region inactivated by chronic muscimol infusion. (A)** Representative examples of prefrontal multi-unit recording sites in a vehicle- (top) or muscimol-infused (bottom) animal. Red dots indicate the recording site in which spontaneous activity or injury discharge was observed. Blue dots indicate the recording site in which no activity was observed. Asterisks in the right panels indicate the location of the cannula tip. **(B)** The extent of the inactivated region estimated by multi-unit recording in the representative muscimol-infused animal shown in **(A)**. Blue circles represent the inactivated region. The asterisk in the right panel indicates the location of the cannula tip. **(C)** The extent of the inactivated region estimated by multi-unit recording in each animal. The different color circles represent the inactivated region in individual animals. **(D)** The location of the cannula tip in vehicle- (top) or muscimol-infused (bottom) animals. The different color circles represent the location of the cannula tip in individual animals. Schematic drawings of coronal sections were adapted from Paxinos and Watson (1998) with permission from Elsevier.

To estimate the extent of the area inactivated by chronic muscimol infusion, we performed multi-unit recording in the animals infused with muscimol or vehicle under similar condition of anesthesia and sedation. In a representative animal treated with vehicle, spontaneous activity and injury discharge were recorded in the PFC throughout electrode penetrations (Figure 2A). On the other hand, in a representative animal treated with muscimol, no activity was observed in regions of the ventral (VO) and lateral area (LO) of the OFC. The inactivated region extended approximately 1.0 mm in the anterior and 1.0 mm in the medial and lateral directions from the cannula tip located in the VO (Figure 2B). In the other three muscimol-infused hemispheres, the inactivated regions extended 0.5–1.5 mm in the anterior direction and 0.5–1.0 mm laterally from the cannula tip (Figure 2C). We identified the location of cannula tips in all animals and most of them were found in VO/LO region. The animals in which the cannula tip was located within the VO/LO in both hemispheres were used for data analysis (11 animals in the vehicle group, 14 animals in the muscimol group, Figure 2D). In electric-shock-induced fighting test, we included the pair in which both the cannula was located within VO/LO in either of the animal (seven pairs in the vehicle group, six pairs in the muscimol group).

### Open Field Test and Light-Dark Test

**Figure 3:**
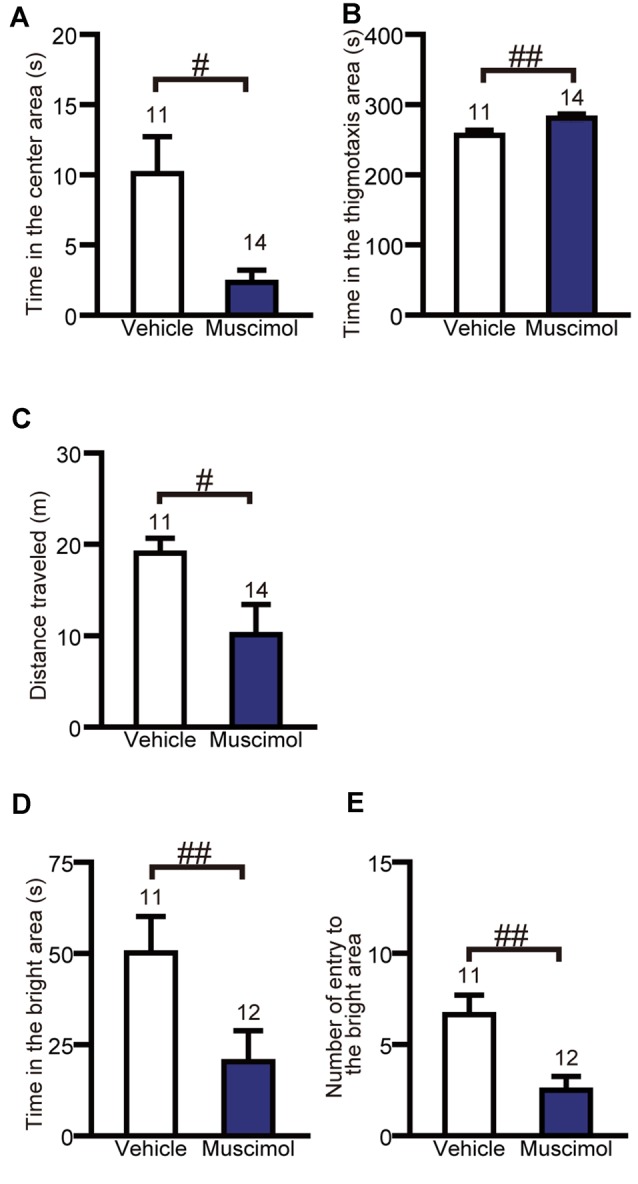
**OFC inactivation increased anxiety-like behaviors.** Time in the center area **(A)**, in the thigmotaxis area **(B)** and distance traveled **(C)** in the open field test. Time in the bright area **(D)** and the number of entries into the bright area **(E)** in the light-dark test. Data are presented as the mean ± S.E. (^#^*P* < 0.05, ^##^*P* < 0.01, Mann-Whitney *U*-test). The number of animals in each group is given above the error bar.

To evaluate the effect of chronic VO/LO inactivation on anxiety-like behavior, we performed the open field test and light-dark test. In the open field test, muscimol treatment significantly decreased the time spent in the center area and total distance traveled and increased the time spent in the thigmotaxis area compared to the animal treated with vehicle (time in the center area: *U*_(11,14)_ = 32.000, *P* = 0.013, total distance traveled: *U*_(11,14)_ = 32.000, *P* = 0.013, time in the thigmotaxis area: *U*_(11,14)_ = 27.000, *P* = 0.005, Mann-Whitney *U-test*, Figures 3A–C). In the light-dark test, muscimol treatment significantly decreased the time spent in the bright area and the number of entries to the bright area compared to the vehicle group (time spent in the bright area: *U*_(11,12)_ = 20.000, *P* = 0.004, number of entries to the bright area: *U*_(11,12)_ = 15.000, *P* = 0.001, Mann-Whitney *U*-test, Figures 3D,E). These results indicate that the inactivation of the VO/LO increased anxiety-like behaviors.

### Forced Swim Test

**Figure 4:**
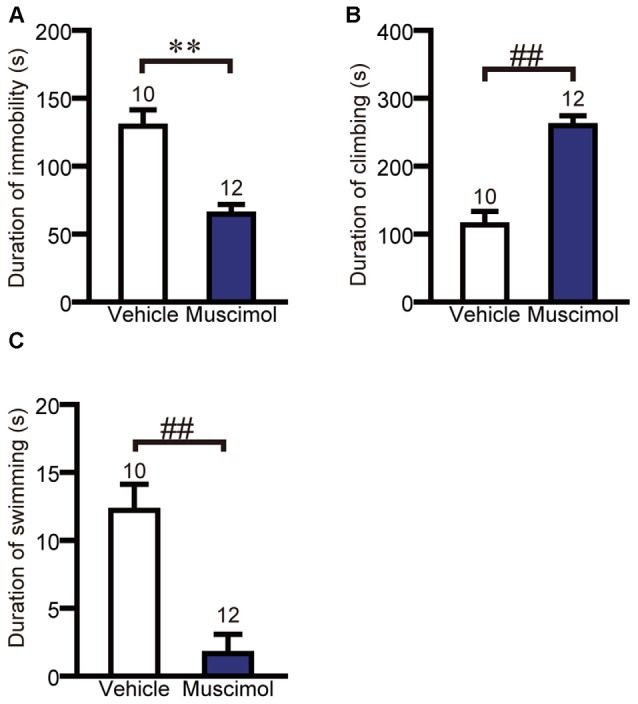
**OFC inactivation attenuated depression-like behavior.** The duration of immobility **(A)**, climbing **(B)** and swimming **(C)** in the forced swim test. Data are presented as the mean ± S.E. (**P < 0.01, unpaired *t*-test, ^##^*P* < 0.01, Mann-Whitney *U*-test). The number of animals in each group is given above the error bar.

We examined the effect of VO/LO inactivation on depression-like behavior in the forced swim test. In the test session, muscimol treatment significantly decreased the duration of immobility and swimming, and increased the duration of climbing compared to the vehicle group (duration of immobility: *F*_(1,20)_ = 23.013, *p* < 0.001, unpaired *t*-test, duration of climbing: *U*_(10,12)_ = 7.000, *P* < 0.001, Mann-Whitney *U*-test, duration of swimming: *U*_(10,12)_ = 9.000, *P* < 0.001, Figure 4). These results indicate that the inactivation of the VO/LO attenuated depression-like behavior.

### Electric-Shock-Induced Fighting Test

**Figure 5:**
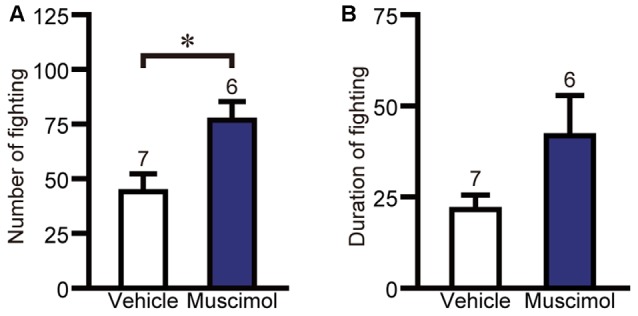
**OFC inactivation enhanced impulsive aggression.** The number **(A)** and duration **(B)** of fighting behavior in the electric-shock-induced fighting test. Data are presented as the mean ± S.E. (*P < 0.01, unpaired *t*-test). The number of animals in each group is given above the error bar.

The effect of VO/LO inactivation on impulsive fighting was evaluated using the electric-shock-induced fighting test. In the test, muscimol treatment increased the number of fighting behaviors compared to the vehicle group (*F*_(1,11)_ = 7.667, *P* = 0.018, unpaired *t*-test, Figure 5A), while there was no significant difference between the two groups in the duration of fighting behavior (*F*_(1,11)_ = 3.367, *P* = 0.133, unpaired *t*-test, Figure 5B). These results indicate that orbitofrontal inactivation enhanced impulsive aggression.

### Plasma Level of Corticosterone After Restraint Stress

**Figure 6:**
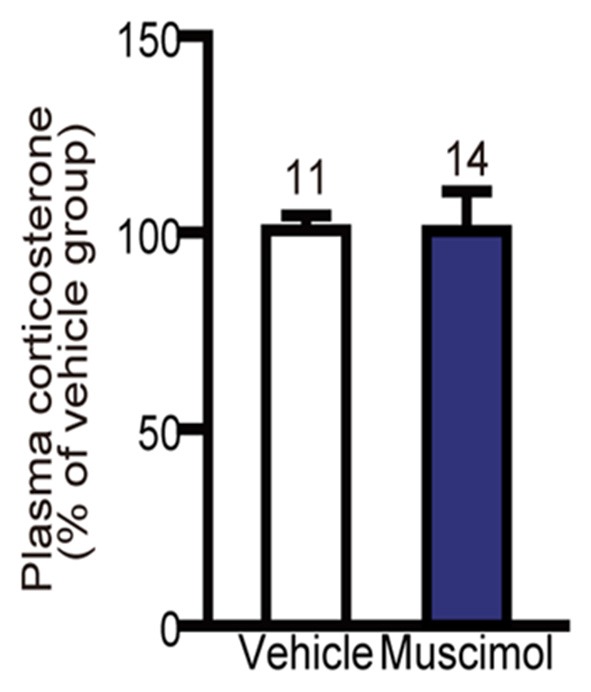
**Effect of OFC inactivation on endocrine response to stress.** Plasma corticosterone level after restraint stress. There was no significant difference between the two groups (Mann-Whitney *U*-test). Data are normalized to the mean value of the vehicle group and presented as the mean ± S.E. The number of animals in each group is given above the error bar.

In the present study, we found no significant difference in the plasma level of corticosterone following 30 min restraint stress between the muscimol and vehicle groups (*U*_(11,14)_ = 59.000, *P* = 0.344, Mann-Whitney *U*-test, Figure 6). These data suggest that the VO/LO in the rat is not involved in the regulation of corticosterone levels in response to restraint stress.

## Discussion

In this study, we showed that chronic inactivation of the ventral and lateral part of the OFC (VO/LO) increased anxiety-like behavior in the open-field test and light-dark test, as well as impulsive aggression in the electric-shock-induced fighting test. On the other hand, it reduced depression-like behavior in the forced swim test and did not cause a significant change in corticosterone secretion in response to restraint stress.

### OFC Dysfunction and Anxiety-Like Behavior in Rodents

The result in the present study, chronic inactivation of the VO/LO increased anxiety-like behaviors, suggests that the VO/LO have an inhibitory role against anxiety-like behaviors in rodents. In a previous study, transient inactivation of the medial part of the OFC by lidocaine infusion increased the anxiety-like defensive response of mice when they were exposed to rats (Wall et al., 2004). On the other hand, excitotoxic lesion of the entire OFC, LO or VO/LO did not alter anxiety-like behavior of rats evaluated using the open field test, elevated plus maze test and successive alley test (modified elevated plus maze test; Lacroix et al., 2000; Rudebeck et al., 2007; Orsini et al., 2015). This inconsistency may have arisen from the differences in the manipulation (pharmacological inactivation or lesion), the duration of OFC dysfunction (acute or chronic) or the size and location of the targeted area (small or large, medial or lateral part of OFC).

Regarding the duration of OFC dysfunction, animal behavior was examined within several minutes after starting inactivation in the acute study (Wall et al., 2004), while the lesion studies (Lacroix et al., 2000; Rudebeck et al., 2007; Orsini et al., 2015) examined behavior following a period of 1 week or more after making the OFC lesion. Thus, it is possible that the effect of OFC inactivation is rescued within the following several days and therefore not found in the lesion studies. However, the present results show that chronic inactivation of the VO/LO can affect anxiety-like behavior.

As to the methods for OFC inactivation, the previous studies used excitotoxic lesion or pharmacological inactivation using lidocaine. Because they could cause unintended damage to other brain regions connected anterogradely or retrogradely (Vanburen, 1963; Poduri et al., 1995) and to passing fibers, respectively, the effect of the lesion and inactivation might not have been restricted to the OFC. On the other hand, in the present experiments, we have inactivated the OFC using muscimol, which selectively inhibits somatic action potentials and exerts fewer effects on axons because of the sparse density of axonal GABA_A_ receptors (Martin and Ghez, 1999; Robbins et al., 2013). Therefore, the present results demonstrate that chronic inactivation restricted to the VO/LO can augment anxiety-like behavior in rats.

Several previous studies focused on another prefrontal region, the medial PFC (mPFC), and found that inactivation or lesion of the entire mPFC consistently attenuated anxiety-like behaviors in rodents (Lacroix et al., 2000; Sullivan and Gratton, 2002; Deacon et al., 2003; Shah and Treit, 2003; Blanco et al., 2009; Solati et al., 2013). The OFC and mPFC might have counteracting functions in the regulation of anxiety-like behavior in rodents.

### OFC Facilitates Depression-Like Behavior in Rodents

To our knowledge, this is the first study to demonstrate the effect of OFC inactivation on depression-like behavior in rodents. Muscimol infused animals significantly reduced immobility and increased climbing behavior. Swimming behavior was also reduced probably because these animals had spent time in climbing behavior throughout the test period. Thus, the inactivation of the VO/LO significantly suppressed depression-like behavior in the forced swim test, suggesting a facilitatory role of the VO/LO in depression-like behavior. A previous study reported that the microinjection of a histone deacetylases inhibitor, valproic acid into the VO/LO showed antidepressant-like effects in the forced swim test (Xing et al., 2011). Although valproic acid might have exerted the antidepressant-like effects through epigenetic mechanisms, it is known that valproic acid acts as a GABA enhancer by inhibiting GABA transaminase (Johannessen, 2000). Therefore, the antidepressant-like effects of valproic acid injection may reflect a suppression of neural activity in the VO/LO as observed in the present experiments.

Other studies reported that inactivation or blockade of NMDA receptor in the ventral subdivision of the mPFC (infralimbic cortex, IL) reduced depression-like behavior (Scopinho et al., 2010; Slattery et al., 2011; Pereira et al., 2015) in rats. Thus, both the VO/LO and IL might have a facilitating function for depression-like behavior.

### OFC Dysfunction Increases Impulsivity and Aggression in Rodents

Previous studies have shown that electric lesion of the OFC enhanced impulsive aggression in the electric-shock-induced fighting test used in the present experiments (Kolb, 1974; Kolb and Nonneman, 1974). Other studies also reported that lesion of the entire OFC or LO increased impulsivity and aggression in rats (de Bruin et al., 1983; Rudebeck et al., 2007; Mar et al., 2011). These findings suggest a role of the OFC in the regulation of impulsivity and aggression, though it is not conclusive because lesion experiments always carry a possibility that other brain regions might be affected due to cell loss and damage of passing fibers. In fact, in a recent study (Takahashi et al., 2014), optogenetic activation of principal neurons in the mPFC suppressed aggressive behavior of mice, but the activation was not effective when given to the OFC. On the other hand, the present study using local inactivation by muscimol provides further evidence for a role of the VO/LO in the regulation of impulsivity and aggression. Activity in the VO/LO might be necessary but not sufficient for the suppression of aggressive behavior.

### OFC Inactivation Did Not Affect Endocrine Response to Stress in Rats

Human functional imaging studies reported that activity in the OFC and mPFC correlates with an increase and decrease of cortisol secretion, respectively (Dedovic et al., 2009). Moreover, electrical stimulation of the OFC increased blood cortisol levels in rhesus monkeys (Hall and Marr, 1975). In rats, OFC stimulation elicited defensive and escape reaction accompanied with blood adrenocorticotropic hormone (ACTH) level (Endroczi et al., 1958). On the other hand, in the present study, chronic inactivation of the VO/LO did not alter the plasma corticosterone level in response to restraint stress in rats. In rodents, the ventral mPFC (IL) is functionally homologous to the primate orbitomedial PFC, with both being autonomic centers (Vertes, 2004), and IL lesions decreased corticosterone secretion in response to restraint stress (Radley et al., 2006). Therefore, IL dependent pathway rather than OFC dependent pathway may regulate secretion of corticosterone in response to restraint stress. The previous results demonstrating the secretion of ACTH by OFC stimulation (Endroczi et al., 1958) suggest that OFC dependent pathway may mediate the effect of other kind of stress. Alternatively, it is possible that the critical region for corticosterone secretion is restricted to the IL rather than the OFC, and the results by OFC stimulation might be an off-target effect by stimulation of passing fibers to the IL.

In the present study, the plasma corticosterone level was measured following a battery of behavioral tests over 5 days. Previous studies demonstrated that repeated daily restraint stress weakened an increase of plasma corticosterone in response to restraint stress possibly through habituation to stress experience (Cole et al., 2000; Girotti et al., 2006; Grissom et al., 2007). These findings raise a possibility that a battery of behavioral tests in the present study might have induced habituation to stress and affected the plasma corticosterone response. On the other hand, successive daily exposure to novel stress did not induce a weakening of plasma corticosterone response (Marin et al., 2007). In the present experiments, the animals had been exposed to a battery of distinct behavioral tests over 5 days. Therefore, it is not plausible that habituation to repeated stress had weakened the plasma corticosterone response in the present experiments, although we can not exclude the possibility that the stress in behavioral tests might had affected the response in plasma corticosterone level and made it difficult to discriminate the muscimol-treated and control animals.

### Neuronal Mechanism of Orbitofrontal Regulation of Affective Behaviors

The present study demonstrated that VO/LO inactivation enhanced anxiety-like and aggressive behaviors and suppressed depression-like behavior at the same time. The VO/LO sends projection to several brain structures which are involved in emotional behavior, such as mPFC, amygdala, striatum, hypothalamus, raphe and ventral tegmental area (VTA; Hoover and Vertes, 2011). One possibility is that OFC might have modulated affective behaviors through one of those structures in the present study. For example, dopaminergic system is considered to be important in all of anxiety- and depression-like, and aggressive behaviors. Although there is no study investigating the effect of dopaminergic manipulation on these behaviors at the same time, pharmacological studies showed that dopamine receptor agonist or dopamine reuptake inhibitor exerted anxiogenic effects in the open field test and light-dark test in mice (Simon et al., 1993, 1994). On the other hand, dopamine reuptake inhibitor reduced depression-like behavior in the forced swim test in rats (Hemby et al., 1997). Moreover, intraventricular administration of dopamine enhanced impulsive aggression in the electric-shock-induced fighting test in rats (Geyer and Segal, 1974). The OFC sends direct projections to the VTA which is the origin of dopaminergic neurons (Vázquez-Borsetti et al., 2009; Hoover and Vertes, 2011), and electrical stimulation of the OFC inhibits activity of the majority of dopaminergic neurons in the VTA (Lodge, 2011; Takahashi et al., 2011). Therefore, the chronic inactivation of the VO/LO might lead to these behavioral changes via activation of dopaminergic modulation. Alternatively, OFC inactivation might have altered each behavior through distinct downstream circuit. For instance, the basolateral complex of amygdala (BLA) is implicated in the expression of anxiety-like and aggressive behaviors in rodents (Eichelman, 1971; Tye et al., 2011). The LO sends projection to the intercalated nuclei of amygdala, which exerts an inhibitory influence on the BLA (Rempel-Clower, 2007). Hence, the OFC inactivation might cause an enhancement of anxiety-like and aggressive behaviors through disinhibition of the amygdala. Also, OFC sends projection to the dorsal raphe and ventral striatum (Hoover and Vertes, 2011) which are involved in the regulation of depression-like behavior (Russo and Nestler, 2013; Teissier et al., 2015), thus OFC inactivation might alter depression-like behavior through these structures. The projection specific manipulation of neural activity will address these possibilities in the future.

Serotonin (5-HT) has been considered to influence affective states and emotional processing such as anxiety, depression, impulsivity and aversive processing (Cools et al., 2008; Coccaro et al., 2011; Albert et al., 2014). While the OFC may regulate serotonergic system through its projection to the raphe, several studies reported that serotonergic modulation in the OFC has an important role in the OFC dependent functions such as reversal learning and response inhibition (Walker et al., 2006; Boulougouris et al., 2008; West et al., 2013). Although the contribution of 5-HT in the OFC to the emotional behavior such as anxiety- and depression-like behavior is less understood in rodents, it is reported that microinjection of 5-HT_1A_ or 5-HT_1B_ receptors agonist into the OFC suppressed aggressive behavior in mice (De Almeida et al., 2006; Centenaro et al., 2008; Stein et al., 2013). Also, systemic administration of 5-HT_1A_ receptor agonist or 5-HT reuptake inhibitor enhanced the firing of principal neurons and the expression of c-fos in the PFC (Hajós-Korcsok and Sharp, 1999; Jongsma et al., 2002; Lladó-Pelfort et al., 2012). Therefore, 5-HT may regulate affective behaviors via modulation of intrinsic neural activity in the OFC in addition to mediating the information from the OFC.

### Comparison With Studies in Old World Monkeys and Humans

Contribution of OFC to emotional behavior has been studied in macaque monkey. Aspirative lesion of the OFC was reported to increase aggressive behavior to human intruder, consistent with the present results in rats, while showing a decrease in fear response to snake (Izquierdo et al., 2005; Rudebeck et al., 2006; Kalin et al., 2007). However, these findings are not conclusive because excitotoxic lesion of the OFC did not alter the fear response (Machado et al., 2009; Noonan et al., 2010; Rudebeck et al., 2013). Moreover, OFC strip lesion, which mimics the damage caused by aspiration to passing fibers, replicated the fear decreasing effect (Rudebeck et al., 2013). Therefore, the effect of OFC inactivation in macaque is not conclusive yet and needs further study using more refined targeting method to consider the role of OFC subregions and possible difference between animal species.

In human imaging studies, a lesion of the OFC or ventromedial PFC (vmPFC, which includes OFC) caused abnormal anxiety, edginess, impulsivity and aggression (Grafman et al., 1986, 1996; Berlin et al., 2004, 2005), and hypoactivity of the OFC was reported in patients with social anxiety disorder and borderline personality disorder with impulsive aggression (Soloff et al., 2003; Hahn et al., 2011). Moreover, damage of the vmPFC alleviated depression severity (Koenigs et al., 2008; Koenigs and Grafman, 2009), while OFC activity increased in depressive patients (Drevets, 2007). Considering these human imaging studies with our results, the OFC may have a suppressive function in anxiety and impulsive aggression and a facilitative function with depressive symptoms in both humans and rodents.

## Author Contributions

HK and SI conceived and designed the experiments. HK, SM, EF and RH performed surgery and the behavioral experiments. HK analyzed the data. HK, SI and YH contributed to writing the manuscript.

## Funding

This work was supported by MEXT KAKENHI Grant Number JP15H01440 and JSPS KAKENHI Grant Number JP16K13111 to YH.

## Conflict of Interest Statement

The authors declare that the research was conducted in the absence of any commercial or financial relationships that could be construed as a potential conflict of interest.
